# Hemoglobin Providence (β82 Lys > Asn, Asp) and lower‐than‐expected HbA1c in a nonadherent teenager with type 1 diabetes: a case report and literature review

**DOI:** 10.1002/ccr3.1244

**Published:** 2017-10-30

**Authors:** Charles N. Newman, Christine M. Litwin, Deborah A. Bowlby, Katherine A. Lewis, Remberto C. Paulo

**Affiliations:** ^1^ Department of Pathology and Laboratory Medicine Medical University of South Carolina Charleston South Carolina 29425; ^2^ Division of Pediatric Endocrinology Medical University of South Carolina Charleston South Carolina 29425

**Keywords:** Diabetes, glycated hemoglobin (HbA1c), hemoglobin variant, pediatric endocrinology

## Abstract

Endocrinologists should have a high index of suspicion for a Hb variant when the HbA1c is not consistent with other indices of glycemic control.

## Introduction

The glycated hemoglobin (HbA1c) assay is an indispensable tool in the diagnosis, treatment, and monitoring of patients with diabetes. However, there are several clinical entities that can interfere with the assay, causing either falsely low or falsely high results. Hemoglobin variants, of which over 950 have been described, are capable of interfering with multiple point‐of‐care (POC) and laboratory methodologies 1. It is estimated that more than 150,000 patients with diabetes in the United States have a hemoglobin variant, and this prevalence may be as high as one‐third in other parts of the world 2. We report a case of falsely low HbA1c in a patient with a rare hemoglobin (Hb) variant, Hb Providence. To our knowledge, this is the first report of this condition causing interference with the HbA1c assay. We also present a summary of other beta chain Hb variants leading to falsely low HbA1c readings from POC immunoassays reported in the literature.

## Methods

The patient was diagnosed with type 1 diabetes when he presented at 10 years of age with polyuria, polydipsia, nocturia, weight loss, and blood glucose of 796 mg/dL. POC HbA1c (DCA Vantage Analyzer) was 7.2% (55 mmol/mol). The patient was not in ketoacidosis. Pancreatic antibodies (GAD Ab and Islet cell Ab) were positive. He was started on insulin therapy. During subsequent clinic visits, the patient's home glucometer printout showed elevated blood sugars in the 200–300s mg/dL and POC HbA1c in the 6.3–7.1% (45–54 mmol/mol) range, suggesting the HbA1c was falsely low. A fructosamine level of 422 *μ*mol/L (normal 200–285 *μ*mol/L) suggested that his HbA1c was greater than 10%. The POC HbA1c continued to be falsely low at 6.4% (46 mmol/mol), so a sample was sent to the chemistry laboratory for HPLC analysis.

## Results

**Figure 1:**
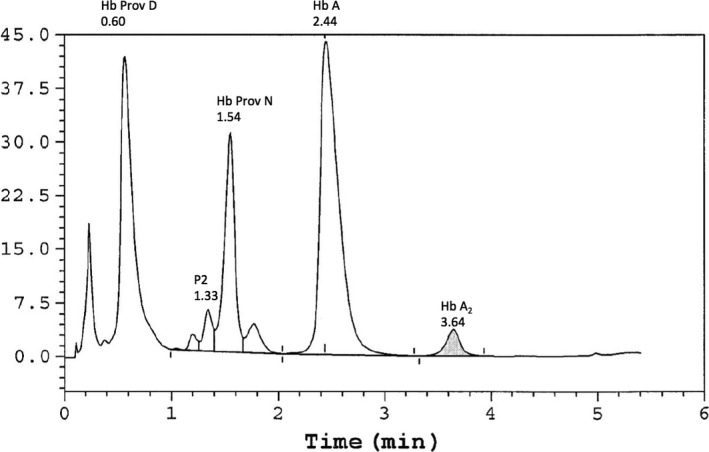
Separation of patient hemolyzate by HPLC Bio‐Rad variant II β‐thal Program. The hemoglobin peaks are labeled along with their retention times.

A variant Hb was observed on the Bio‐Rad Variant II HPLC. Normal HbA and the two forms of Hb Providence were observed. Figure 1 shows the HPLC results of a fresh hemolyzate of the patient's whole blood. HbA was quantitated at 45.6%, HbA2 was quantitated at 2.6%, and the two variants were detected as Hb Providence asparagine (Hb Prov N) at 16.5% with a retention time of 1.54 min and the fast‐eluting‐deaminated Hb Providence aspartic acid (Hb Prov D) at 35.3% with a retention time of 0.60 min. No Hb F was detected.

**Figure 2:**
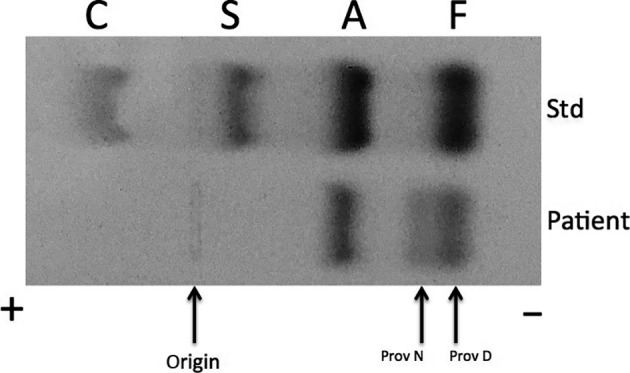
The electrophoretic pattern of Hb Providence N and Providence D on citrate agar (pH 6.2). The zones corresponding to Std show the separation of an artificial mixture of Hb C, S, A, and F, respectively. Arrows point to the origin, Hb Providence N, and Hb Providence D of the patient sample.

Figure 2 demonstrates the electrophoretic results of a fresh hemolyzate on citrate agar (pH 6.2). On citrate agar, three bands are demonstrated: one with a mobility such as Hb A, one with a mobility between Hb A and Hb F, and one with mobility slightly anodic of Hb F.

Bidirectional sequence analysis was performed at Mayo Medical Laboratories in all coding and noncoding portions of the beta Hb gene (*HBB*) with reported mutations. The mutation nomenclature used is based on GenBank accession number NM_000518.4. An alteration was detected at codon 82, AAG>AAC, heterozygous, HGVS: c.249G>C, p.K83N with the classification of the mutation as Hb Providence.

The patient's CBC showed mild erythrocytosis with a Hb of 16.3 g/dL (normal range 11.0–15.0 g/dL) and hematocrit of 48.0% (normal range 35.0–45.0%).

## Discussion

To our knowledge, this is the first report of Hb Providence causing a falsely low HbA1c. Substitution of asparagine for lysine at position 82 in the Hb β‐chain results in Hb Prov N. This asparagine is subsequently deaminated to an aspartic acid in vivo during the life span of the erythrocyte to create Hb Providence D. These changes decrease the affinity of hemoglobin for 2,3‐bisphosphoglycerate, a potent modulator of the affinity of hemoglobin for oxygen. Consequently, this mutation causes the hemoglobin to have moderately high oxygen affinity with subsequent moderate erythrocytosis 3, 4.

Point‐of‐care and laboratory HbA1c measurements can be affected by the presence of a Hb variant. The interference can falsely elevate or decrease the measurement depending upon the variant and the methodology used. Table 1 shows other beta chain Hgb variants reported in the literature to cause falsely low HbA1c using POC immunoassays. The antibodies used in most immunoassays recognize the first four to ten amino acids in the hemoglobin beta chain 2. Hb S, C, Raleigh, and Graz have amino acid substitutions in this region and have been reported to interfere with POC HbA1c immunoassays 5, 6. The amino acid substitution in Hb Rambam, similar to Hb Providence, falls outside of this region but has still been reported to be associated with a decreased HbA1c 6, 7. Further studies are needed to elucidate the mechanisms of interference for Hb Rambam and Providence.

**Table 1:** Summary of β‐chain hemoglobin variants that can give falsely low HbA1c readings using point‐of‐care immunoassays

Variant [references]	Mutation	HbA1c measurement
Hb S 5, 8, 9	β6 Glu > Val	Falsely high
Hb C 5, 6, 8, 10	β6 Glu > Lys	Falsely low or high
Hb Raleigh 1, 6	β1 Val > Ala	Falsely low
Hb Graz 6, 9	β2 His > Leu	Falsely low
Hb Rambam 6, 7	β69 Gly > Asp	Falsely low
Hb Providence^a^	β82 Lys > Asn, Asp	Falsely low

a Current report.

Hb variants may also interfere with laboratory methodologies such as cation exchange chromatography or electrophoresis. In cation exchange chromatography, the native Hb variant may co‐elute with HbA1c, the glycated Hb variant may co‐elute with HbA1c, or the Hb variant may co‐elute with HbA. Comigration can cause increased HbA1c values when electrophoresis or isoelectric focusing is used 2.

Considering the frequency of hemoglobin variants and the numerous ways in which they may interfere with HbA1c measurement, endocrinologists should have a high index of suspicion for a Hb variant when the HbA1c is not consistent with other indices of glycemic control. A blood sample should be sent to the laboratory for HPLC Hb variant analysis. Glycated serum proteins, such as fructosamine and glycated serum albumin, may be used as alternative tests when a patient has a Hb variant that affects HbA1c measurement. Boronate affinity chromatography and electrospray mass spectrometry (ES‐MS) are the laboratory methodologies which demonstrate the least interference from the presence of Hb variants 2.

## Authorship

CNN: wrote the initial and revised drafts of this paper and performed the literature review. CML: provided laboratory data, figures, and revision suggestions. DAB, KAL and RCP: provided clinical information and revision suggestions.

## Conflict of Interest

None declared.
